# Dihydromyricetin exhibits potent antibacterial activity and virulence factor inhibitory effects against *Porphyromonas gingivalis*

**DOI:** 10.3389/froh.2026.1872637

**Published:** 2026-08-10

**Authors:** Yanhui Peng, Juan Liu, Qing Liu, Na Liu

**Affiliations:** 1 Hebei Key Laboratory of Stomatology/Hebei Technology Innovation Center of Oral Health, School and Hospital of Stomatology, Hebei Medical University, Shijiazhuang, China; 2 Department of Preventive Dentistry, School and Hospital of Stomatology, Hebei Medical University, Shijiazhuang, China

**Keywords:** antibacterial, chronic periodontitis, dihydromyricetin, mechanism, *Porphyromonas gingivalis*

## Abstract

**Objective:**

Dihydromyricetin has inhibitory effects on various pathogens, but its effect on *Porphyromonas gingivalis* is not yet known. This study investigates the effects of dihydromyricetin on planktonic cells, biofilms, and related virulence factors of *Porphyromonas gingivalis*, evaluates the *in vitro* antibacterial activity of dihydromyricetin against *Porphyromonas gingivalis*, and preliminarily explores its possible antibacterial mechanisms.

**Methods:**

The microdilution method was used to explore the inhibitory effect of dihydromyricetin on planktonic *Porphyromonas gingivalis*. The impact of dihydromyricetin on biofilm formation was assessed via crystal violet staining. The effects on the expression of fimbrial genes (*fimA*, *fimR*, *fimS*) and the capsular polysaccharide gene *PGN-1525* were examined using qRT-PCR. Protein substrate assays were conducted to evaluate the influence of dihydromyricetin on the proteolytic activity of gingipains (arginine protease and lysine protease).

**Results:**

1% DMSO has no significant effect on the growth of *Porphyromonas gingivalis*. MIC of dihydromyricetin against planktonic *Porphyromonas gingivalis* was 160 µg/mL, while the MBC was 320 µg/mL. DMY at 160–640 µg/mL downregulated fimbrial (*fimA*, *fimR*, *fimS*) and capsular polysaccharide (*PGN_1525*) gene expression; 40–160 µg/mL DMY concentration-dependently suppressed Rgp and Kgp proteolytic activity.

**Conclusion:**

Dihydromyricetin has shown relatively significant antibacterial activity and virulence factor inhibition against *Porphyromonas gingivalis*.

## 1 Introduction

Chronic periodontitis is a common inflammatory and destructive periodontal disease. *Porphyromonas gingivalis* (*P. gingivalis*) is one of the major pathogenic bacteria causing chronic periodontitis (1). It colonizes the oral cavity for a long time and forms biofilms through the synergistic action of various virulence factors such as fimbriae, gingipains, and capsules (2–4), thereby directly invading and damaging periodontal tissues (5). It can also trigger excessive immune-inflammatory responses in the host and cooperate with other oral pathogenic bacteria to promote the occurrence and development of chronic periodontitis (6). Therefore, eliminating *P. gingivalis* or attenuating its virulence through antibacterial therapy is one of the important approaches for the clinical treatment of chronic periodontitis.

Currently, clinical methods for treating chronic periodontitis mainly include periodontal basic treatment (supragingival scaling, subgingival scaling, and root planing) and periodontal surgical treatment (7). However, there are some infected sites in periodontal lesions that are difficult to reach with instruments, and microorganisms from other parts of the oral cavity may invade periodontal tissues or re-colonize the periodontal pocket. Therefore, adjuvant antibacterial drug therapy is often used (8), especially for patients with systemic diseases or those who cannot effectively manage oral hygiene. Antibacterial drugs play a crucial role in controlling periodontal infections and preventing complications.

Traditional antibacterial drugs, such as nitroimidazoles, tetracyclines, β-lactams, and macrolides (9), have been used in clinical treatment. However, due to various factors, the limitations of antibiotic use have become increasingly prominent, among which drug resistance is the most concerning. Studies have shown that the sensitivity of *P. gingivalis* to various antibiotics has decreased (8, 10). In this context, there is an urgent need to find more effective antibacterial drugs or active ingredients. In this regard, natural plant extracts show unique advantages. Existing evidence indicates that various natural plant extracts have clear antibacterial effects and are believed to have different targets of action from traditional antibiotics (11–13). The resistance mechanisms established by bacteria against traditional antibiotics cannot effectively act on them, so natural plant extracts are not prone to inducing drug resistance (14). In addition, natural plant extracts have the advantages of wide sources, low cost, few side effects on the human body, and easy acceptance by patients (15), showing broad application prospects.

Vine tea, as a traditional Chinese herbal medicine with a history of more than 1,500 years, its main active ingredient is dihydromyricetin (DMY) (16). As a plant-derived flavonoid compound (17), DMY has been found to have inhibitory effects on various common pathogenic bacteria, such as *Vibrio parahaemolyticus*, *Staphylococcus aureus*, *Escherichia coli*, *Salmonella paratyphi*, *Pseudomonas aeruginosa*, *Bacillus subtilis*, and *Candida albicans*, showing broad-spectrum antibacterial activity (18–22). Previous mechanistic studies confirm DMY exerts bactericidal effects by disrupting bacterial membrane permeability, elevating intracellular reactive oxygen species, inhibiting metabolic enzymes and interfering with microbial nucleic acid replication. Therefore, this study aimed to evaluate the inhibitory effect of DMY on planktonic cells and biofilms of *P. gingivalis*, analyze its impact on related virulence factors, and explore its possible antibacterial mechanisms, in order to provide experimental basis for the development of new drugs for chronic periodontitis.

## 2 Materials and methods

### 2.1 Main reagents and instruments

DMY (purity ≥98%) was purchased in the form of white crystalline powder from Beijing Solarbio Science & Technology Co., Ltd., China; *P. gingivalis* ATCC 33277 (Shanghai Huzheng Biotechnology Co., Ltd., China; strain identification was performed by Shanghai Sangon Biotech Co., Ltd. The 16S rDNA full-length sequence of the strain was amplified by PCR and sequenced, and the sequencing results were compared with BLAST in the NCBI database to determine the strain species); DMSO (Beijing Solarbio Science & Technology Co., Ltd., China); Brain Heart Infusion Broth (BHI) (Qingdao Haibo Biotechnology Co., Ltd., China); Bacterial RNA Extraction Kit (Beijing Aidlab Biotechnologies Co., Ltd., China); Anaerobic gas-generating packs, anaerobic culture jars (Mitsubishi, Japan); Biosafety cabinet (Heal Force Model: A2, China); Constant temperature incubator (Shanghai Xinmiao Model: DNP-9052BS-Ⅲ, China); High-pressure steam sterilizer (Shandong Xinhua Medical Model: LMQ.C, China); McFarland nephelometer (Shanghai Meihua Medical Co., Ltd., China); Water bath shaker incubator (Shanghai Zhichu Instrument Co., Ltd. Model: ZHSY-50ES, China); Full-wavelength microplate reader (MDC Model: VERSA max, USA).

### 2.2 Experimental methods

#### 2.2.1 Preparation of bacterial suspension and biofilm culture conditions

Cryopreserved *P. gingivalis* ATCC 33277 was streaked onto Columbia blood agar with 5% defibrinated sheep blood and cultured anaerobically at 37 °C for 14 days in jars filled with 80% N_2_, 10% H_2_, 10% CO_2_. Single colonies were cultured in supplemented BHI broth containing hemin (5 μg/mL) and menadione (1 μg/mL). Log-phase suspension was standardized to 0.5 McFarland (1.5 × 10^8^ CFU/mL). For biofilm tests, static incubation for 48 h was applied without shaking. Preformed biofilms were cultured for 48 h, rinsed with PBS before drug treatment and incubated for another 48 h.

#### 2.2.2 Effect of different concentrations of DMSO on *P. gingivalis*

The microdilution method combined with optical density (OD) value was used to evaluate the effect of different concentrations of DMSO on *P. gingivalis*. 100 µL of 2× working concentration DMSO solutions with concentrations of 4%, 3%, and 2% prepared in advance and 100 µL of 2× working bacterial suspension were added to 96-well plates, so that the final concentrations of DMSO in the experimental groups were 2%, 1.5%, and 1%, respectively. The plates were anaerobically cultured at 37 °C for 48 h in a constant temperature incubator (22). A blank control group without DMSO was also set up, and each concentration had 3 replicate wells. The effect of DMSO on *P. gingivalis* was expressed as the growth inhibition rate (GI) (%):

$$\mathrm{GI}(\text{\%})=\frac{A1-A2}{A2-A0}\times 100\text{\%}$$

Where GI (%) represents the growth inhibition rate of DMSO on *P. gingivalis*; A0 represents the OD_600_ value of the blank control group; A1 represents the OD_600_ value of the growth control group; A2 represents the OD_600_ value after treatment with different concentrations of DMSO for 48 h.

#### 2.2.3 Effect of DMY on planktonic *P. gingivalis*

DMY was serially diluted using the microdilution method and added to 96-well plates, mixed with 100 μL of *P. gingivalis* bacterial suspension at 1.5 × 10^8^ CFU/mL, so that the final concentrations of DMY were 640 µg/mL, 320 µg/mL, 160 µg/mL, 80 µg/mL, and 40 µg/mL. The plates were anaerobically cultured at 37 °C for 48 h in a constant temperature incubator (23). A blank control group without DMY was also set up, and each concentration had 3 replicate wells. The lowest drug concentration at which no bacterial growth was observed with the naked eye (i.e., the liquid in the microplate was clear and free of turbidity) was recorded as the minimum inhibitory concentration (MIC). 10 µL of culture solution was randomly selected from one well of each column and inoculated on a blood agar plate, which was anaerobically cultured at 37 °C for 48 h in a constant temperature incubator. The lowest drug concentration at which no bacterial growth was observed on the blood agar plate was recorded as the minimum bactericidal concentration (MBC).

#### 2.2.4 Effect of DMY on *P. gingivalis* biofilms

##### 2.2.4.1 Detection of the inhibitory effect of DMY on *P. gingivalis* biofilm formation by crystal violet staining

*P. gingivalis* bacterial suspension (100 μL per well) was mixed with different concentrations of DMY (100 μL per well) and anaerobically cultured at 37 °C for 48 h in a constant temperature incubator (24). A growth control group (100 µL BHI liquid medium and 100 µL bacterial suspension) and a blank control group (200 µL BHI) were also set up. Crystal violet staining was performed, and the absorbance value was measured at 590 nm using a microplate reader. The minimum biofilm inhibition concentration (MBIC) was defined as the lowest drug concentration that inhibited 50% or 90% of biofilm formation compared with the control group, i.e., MBIC_50_ and MBIC_90_ (25). Each concentration had 3 replicate wells, and the experiment was repeated three times. The biofilm formation inhibition rate was calculated as follows: Growth inhibition rate = [(A590 nm of growth control group − A590 nm of experimental group)/(A590 nm of growth control group − A590 nm of blank control group)] × 100%.

##### 2.2.4.2 Detection of the eradication effect of DMY on mature *P. gingivalis* biofilms by crystal violet staining

*P. gingivalis* bacterial suspension (200 μL per well) was anaerobically cultured at 37 °C for 48 h in a constant temperature incubator (24). After washing off planktonic bacteria with PBS, 200 μL of DMY serially diluted by the microdilution method with final concentrations of 640 µg/mL, 320 µg/mL, 160 µg/mL, 80 µg/mL, and 40 µg/mL was added. A growth control group (100 µL BHI liquid medium and 100 µL bacterial suspension) and a blank control group (200 µL BHI) were also set up. After further anaerobic culture for 48 h (24), crystal violet staining was performed, and the absorbance value was measured at 590 nm using a microplate reader. The minimum biofilm eradication concentration (MBEC) was defined as the lowest drug concentration that eradicated 50% of biofilm formation compared with the control group, i.e., MBEC_50_ (25). Each concentration had 3 replicate wells, and the experiment was repeated three times. The biofilm eradication rate was calculated as follows: Growth eradication rate = [(A590 nm of growth control group − A590 nm of experimental group)/(A590 nm of growth control group − A590 nm of blank control group)] × 100%.

#### 2.2.5 Effect of DMY on *P. gingivalis* virulence factors

##### 2.2.5.1 Detection of the effect of DMY on the expression of *P. gingivalis* fimbrial genes (*fimA*, *fimR*, *fimS*) by qRT-PCR

DMY was serially diluted using the microdilution method and added to 96-well plates, mixed with 100 μL of *P. gingivalis* bacterial suspension at 1.5 × 10^8^ CFU/mL, so that the final concentrations of DMY were 640 µg/mL, 320 µg/mL, 160 µg/mL, 80 µg/mL, and 40 µg/mL. The plates were anaerobically cultured at 37 °C for 48 h in a constant temperature incubator. Total bacterial RNA was extracted following the standard protocol of the Aidlab bacterial RNA extraction kit, including lysozyme bacterial lysis, chloroform phase separation, isopropanol precipitation and 75% ethanol washing steps. RNA was strictly quality-checked prior to reverse transcription. RNA purity was assessed by ultraviolet spectrophotometry based on the A260/A280 ratio, and values between 1.8 and 2.1 were defined as qualified, while RNA integrity was verified via 1% agarose gel electrophoresis. For reverse transcription, 500 ng qualified total RNA was subjected to genomic DNA removal, followed by cDNA synthesis under the incubation program: 42 °C for 15 min, then 85 °C for 5 s to terminate the reaction. All primers targeting the virulence-related genes *fimA*, *fimR*, *fimS*, and *PGN_1525*, as well as the reference gene 16S rRNA, were synthesized by Sangon Biotech (Shanghai, China). Each primer pair was pre-validated by conventional PCR and agarose gel electrophoresis, and only primers producing a single specific band without non-specific amplification were applied for formal qRT-PCR. To guarantee the accuracy of quantitative detection, cDNA was serially diluted at a 5-fold gradient to generate standard curves, and the amplification efficiency calculated from the slope of each curve ranged from 90% to 110% for all primers, fully satisfying the quantitative requirements. The 16S rRNA gene of *P. gingivalis* was used as the internal reference to correct sample loading differences, and the relative expression levels of target genes were calculated using 2^−△△Ct^ method with the gene expression level of the control group set as the normalization baseline. The primer sequences are shown in Table 1.

**Table 1:** Specific primer sequences for *P. gingivalis* fimbrial genes *fimA*, *fimR*, and *fimS*.

Gene	Primer	Sequence	Fragment length
*16S*	F	5’-AGGAACTCCGATTGCGAAGGT-3’	140bp
	R	5’-TCGTTTACTGCGTGGACTACC-3’	
*fimA*	F	5’-GGCAGAACCCGTTGTAGAAA-3’	129bp
	R	5’-TGACCTGCATAGACCATGGC-3’	
*fimR*	F	5’-CAATATCTGTGCGATGGCTA-3’	124bp
	R	5’-TGCCAATCCACTAATCCGCT-3’	
*fimS*	F	5’-CGCGACTAACTATCCTGACA-3’	152bp
	R	5’-AGGAATATGCATCGGCTGCA-3’	

The primer sequences are shown in Table 2.

##### 2.2.5.2 Detection of the effect of DMY on the proteolytic activity of *P. gingivalis* gingipains by protein substrate assay

Supernatant collection: Logarithmic-phase bacterial suspensions were centrifuged to collect cell-free culture supernatant for subsequent protease detection. In positive control group, reaction system setup: For DMY treatment groups, 100 µL bacterial supernatant, 50 µL gradient DMY solutions (0, 40, 80, 160 µg/mL), 25 µL DTT, and chromogenic substrates specific for Rgp or Kgp were mixed sequentially. The group without *P. gingivalis* was set as the negative control group. The mixture was incubated in the dark at 37 °C for 4 h, and the absorbance value was measured at 490 nm using a microplate reader. Each concentration had 3 replicate wells, and the experiment was repeated three times.

##### 2.2.5.3 Detection of the effect of DMY on the expression of *P. gingivalis* capsular polysaccharide gene *PGN_1525* by qRT-PCR

The method was the same as 1.2.4.1.

The primer sequences are shown in Table 2.

**Table 2:** Specific primer sequences for *P. gingivalis* capsular polysaccharide gene *PGN_1525*.

Gene	Primer	Sequence	Fragment length
*16S*	F	5’-AGGAACTCCGATTGCGAAGG T-3’	140bp
	R	5’-TCGTTTACTGCGTGGACTACC-3’	
*PGN-1525*	F	5’-GGAGGAGAAAGATTATGTCG-3	196bp
	R	5’-CAAAGAAGCCTCGGACAG-3	

### 2.3 Statistical analysis

Statistical analyses were performed using SPSS 26.0 software. Normally distributed data were presented as mean ± standard deviation ($\bar{x}\pm s$) and compared by one-way analysis of variance (ANOVA) followed by Tukey's *post-hoc* multiple comparison test for pairwise intergroup comparisons. Non-normally distributed data were expressed as median (*P*_25_, *P*_75_) and analyzed via nonparametric rank-sum test. Differences with (*P* < 0.05) were considered statistically significant.

### 2.4 Data statement

All original experimental data are reserved for subsequent unpublished studies of our group and will be deposited into public repositories after the completion of serial article publication.

## 3 Results

### 3.1 Effect of different concentrations of DMSO on *P. gingivalis*

**Figure 1:**
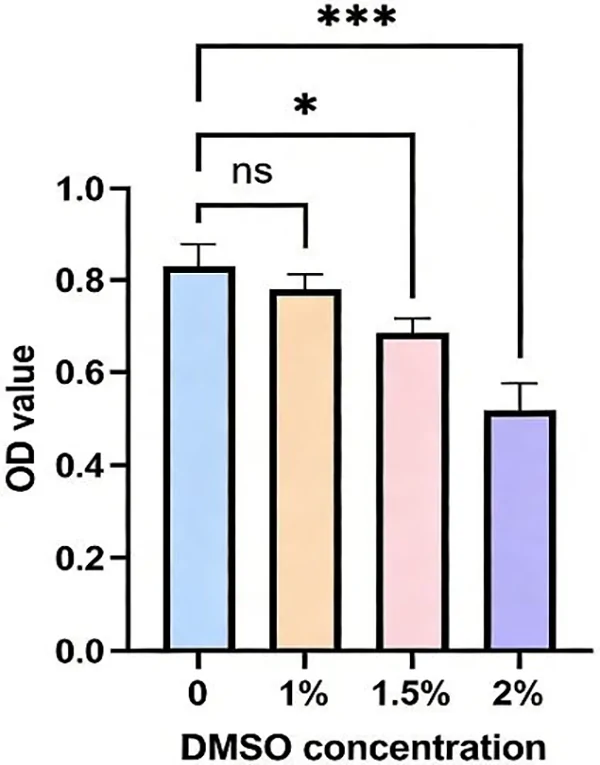
Effect of different concentrations of DMSO on the OD_600_ value of *P. gingivalis*. The microbroth dilution method combined with optical density measurement was employed to investigate the effects of 1%, 1.5%, and 2% of DMSO on the growth of *P. gingivalis*. The results demonstrated that the absorbance at 600 nm (OD_600_) was decreased to varying degrees in all DMSO-treated groups, and the magnitude of the reduction in OD_600_ exhibited a gradual increase with elevating DMSO concentrations. Compared with the concentration of 0, *P < 0.05, ***P < 0.001.

After co-culturing different concentrations of DMSO with *P. gingivalis* for 48 h, the OD_600_ values of each experimental group decreased to varying degrees, and the decrease amplitude gradually increased with the increase of DMSO concentration (Table 3). Statistical analysis showed that there was no significant difference in the OD_600_ value between the 1% DMSO group and the growth control group after 48 h of treatment (*P* > 0.05), while the OD_600_ values of the 1.5% and 2% DMSO groups were lower than those of the growth control group, with statistically significant differences (*P* < 0.05) (Figure 1). This 1% DMSO concentration is only suitable for pure anaerobic bacterial culture in the present study. If mammalian cell co-culture or cytotoxicity assays are performed in subsequent experiments, the final DMSO concentration will be strictly controlled.

**Table 3:** Effect of different concentrations of DMSO on the OD_600_ value of *P. gingivalis*.

DMSO concentration (v/v%)	Mean	Percentile		
		P_25_	P_50_ (median)	P_75_
2	0.514	0.463	0.498	0.573
1.5	0.683	0.651	0.688	0.709
1	0.778	0.762	0.782	0.804
0	0.830	0.828	0.844	0.852

### 3.2 Effect of DMY on planktonic *P. gingivalis* and biofilms

#### 3.2.1 Effect of DMY on planktonic *P. gingivalis*

**Figure 2:**
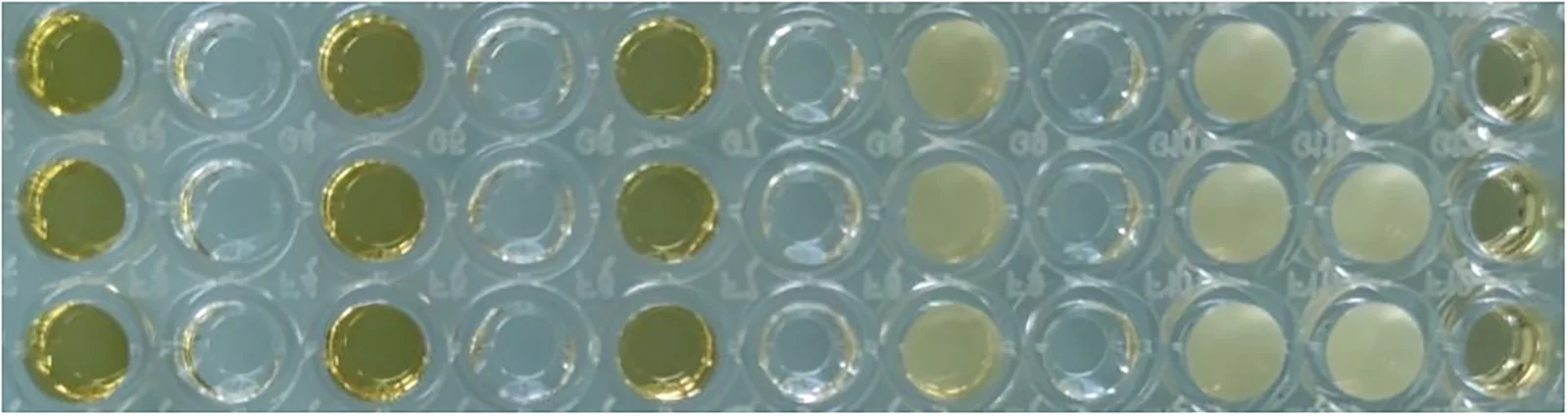
Naked-eye observation of *P. gingivalis* co-incubated with different concentrations of DMY for 48 h. **(A)** 640 µg/mL DMY; **(B)** 320 µg/mL DMY; **(C)** 160 µg/mL DMY; **(D)** 80 µg/mL DMY; **(E)** 40 µg/mL DMY; **(F)** 0 µg/mL DMY; **(G)** blank control group. The microbroth dilution method was used to evaluate the antibacterial effects of different concentrations of DMY on planktonic *P. gingivalis*. Visual inspection revealed that the culture broth was visibly turbid in the 40 µg/mL and 80 µg/mL treatment groups, whereas the broth remained clear and showed no apparent turbidity indicative of bacterial growth in the 160 µg/mL, 320 µg/mL, and 640 µg/mL groups.

**Figure 3:**
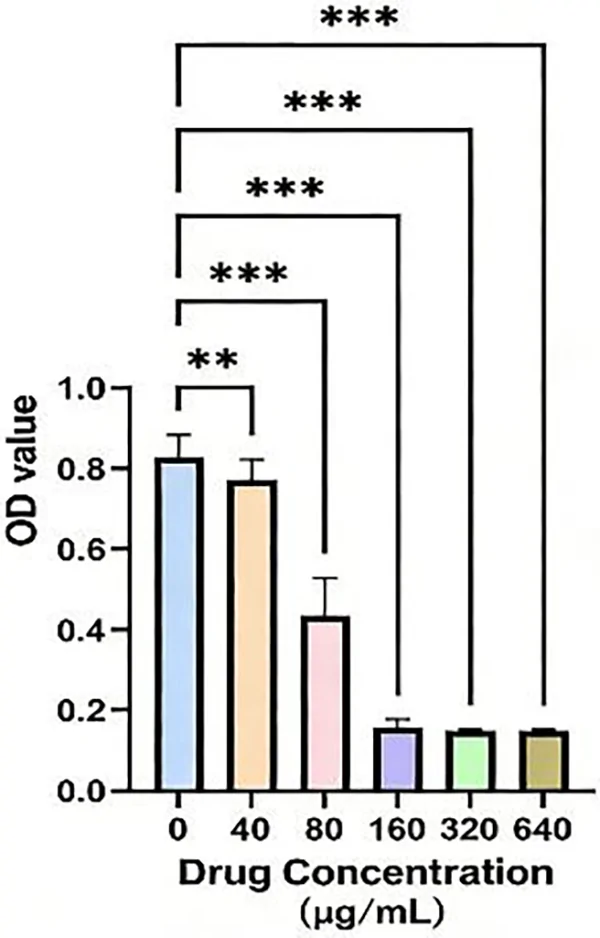
Effect of different concentrations of DMY on the OD_600_ value of planktonic *P. gingivalis*. The effect of DMY at gradient concentrations of 640 µg/mL, 320 µg/mL, 160 µg/mL, 80 µg/mL, and 40 µg/mL on planktonic *P. gingivalis* was evaluated using the broth microdilution method combined with optical density measurement. Compared with the growth control group, the OD_600_ values of all treatment groups were significantly decreased. Compared with the concentration of 0, **P < 0.01, ***P < 0.001.

**Figure 4:**
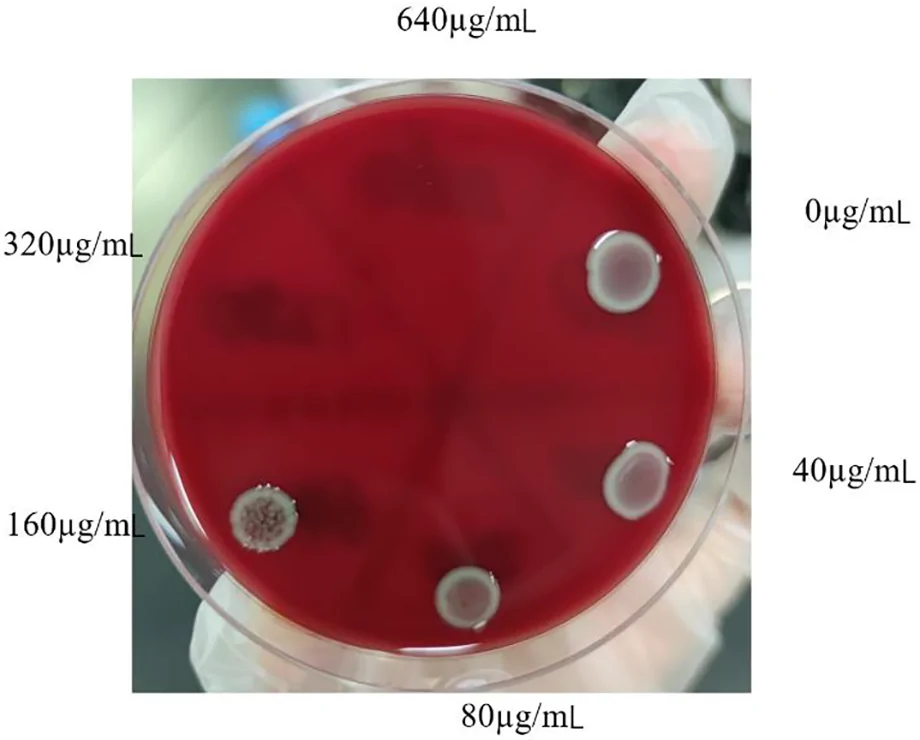
MBC of DMY against planktonic *P. gingivalis*. From each column, 10 µL of culture was randomly aspirated, inoculated onto blood agar, and incubated anaerobically at 37  °C for 48 h before macroscopic observation. Blood agar culture showed that the number of bacterial colonies on blood agar plates gradually decreased with increasing DMY concentration, and no bacterial growth was observed in the 320 µg/mL treatment group.

After treating *P. gingivalis* with different concentrations of DMY for 48 h, the naked-eye observation results showed that the liquid in each well of the 40 µg/mL and 80 µg/mL experimental groups was turbid; while the liquid in each well of the 160 µg/mL, 320 µg/mL, and 640 µg/mL experimental groups was clear, with no obvious turbidity (Figure 2), so the MIC was considered to be 160 µg/mL. The OD_600_ values of each well were measured using a microplate reader, and it was found that compared with the growth control group, the OD_600_ values of the 40 µg/mL, 80 µg/mL, 160 µg/mL, 320 µg/mL, and 640 µg/mL experimental groups were significantly decreased (Table 4), with statistically significant differences (*P* < 0.05) (Figure 3). Blood agar plate culture showed that with the increase of DMY concentration, the number of bacteria on the blood agar plate gradually decreased, and no bacterial growth was observed in the 320 µg/mL experimental group (Figure 4), so the MBC was considered to be 320 µg/mL.

**Table 4:** Effect of different concentrations of DMY on the OD_600_ value of planktonic *P. gingivalis*.

Drug concentration (µg/mL)	Mean	Percentile		
		P_25_	P_50_ (median)	P_75_
640	0.137	0.135	0.137	0.140
320	0.149	0.146	0.150	0.150
160	0.158	0.148	0.151	0.161
80	0.438	0.385	0.441	0.492
40	0.773	0.742	0.762	0.809
0	0.828	0.781	0.851	0.872

#### 3.2.2 Effect of DMY on *P. gingivalis* biofilms

##### 3.2.2.1 Effect of DMY on *P. gingivalis* biofilm formation

**Figure 5:**
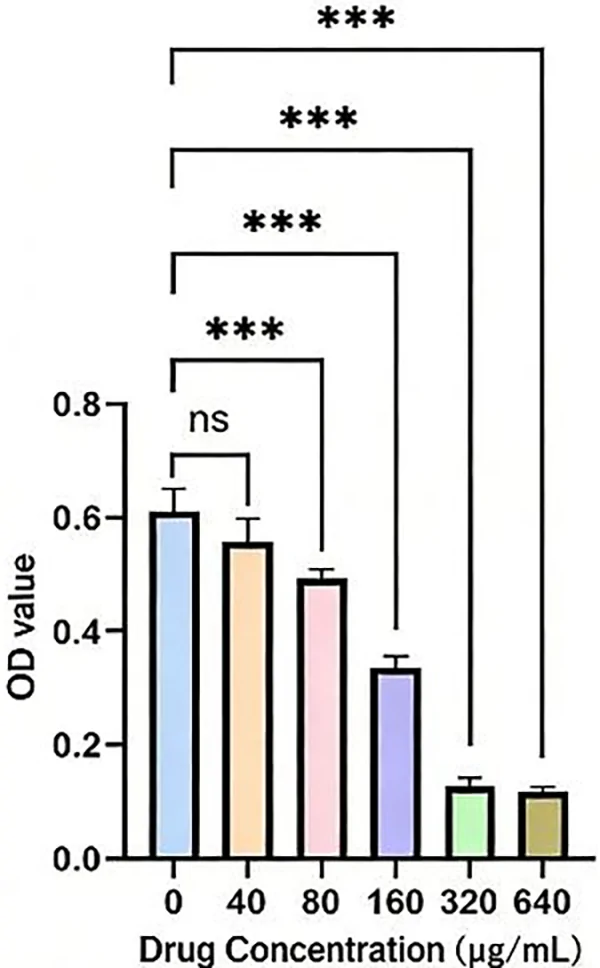
Effect of different concentrations of DMY on *P. gingivalis* biofilm formation. The effect of DMY at concentrations of 640, 320, 160, 80, and 40 µg/mL on *P. gingivalis* biofilm formation was evaluated using the crystal violet staining method combined with optical density measurement. Compared with the growth control group, the OD values of all treatment groups decreased significantly, indicating a marked reduction in biofilm formation. Furthermore, the degree of reduction was positively correlated with DMY concentration. Compared with the concentration of 0, ***P < 0.001.

After co-culturing different concentrations of DMY with *P. gingivalis* bacterial suspension for 48 h, the crystal violet staining method was used to evaluate the effect of DMY on *P. gingivalis* biofilm formation. As shown in Table 5, the OD values of all experimental groups were significantly lower than those of the control group, revealing that dihydromyricetin effectively inhibits *P. gingivalis* biofilm formation in a concentration-dependent manner. Given that crystal violet binds non-specifically to all biofilm constituents including viable bacteria, dead cellular debris and extracellular matrix, the obtained data only reflects changes in total biofilm biomass instead of precise viable cell counts within biofilms. Statistical analysis showed that there was no significant difference between the 40 µg/mL experimental group and the growth control group (*P* > 0.05); while there were statistically significant differences between the 80 µg/mL, 160 µg/mL, 320 µg/mL, 640 µg/mL experimental groups and the growth control group (*P* < 0.05) (Figure 5). The inhibition rate of each concentration of DMY on *P. gingivalis* biofilm formation was calculated, and it was found that with the increase of DMY concentration, the inhibition rate on biofilm formation also showed an upward trend. When the DMY concentrations were 40 µg/mL, 80 µg/mL, 160 µg/mL, 320 µg/mL, and 640 µg/mL, the inhibition rates were 10.19%, 22.07%, 51.70%, 90.94%, and 92.45%, respectively. Therefore, the MBIC_50_ and MBIC_90_ were considered to be 160 µg/mL and 320 µg/mL, respectively.

**Table 5:** Effect of different concentrations of DMY on *P. gingivalis* biofilm formation.

Drug concentration (µg/mL)	Mean	Percentile		
		P_25_	P_50_ (median)	P_75_
640	0.120	0.114	0.118	0.126
320	0.128	0.120	0.134	0.137
160	0.336	0.315	0.338	0.354
80	0.493	0.479	0.501	0.506
40	0.556	0.517	0.557	0.599
0	0.610	0.590	0.604	0.627

### 3.2.2.2 Effect of DMY on mature *P. gingivalis* biofilms

**Figure 6:**
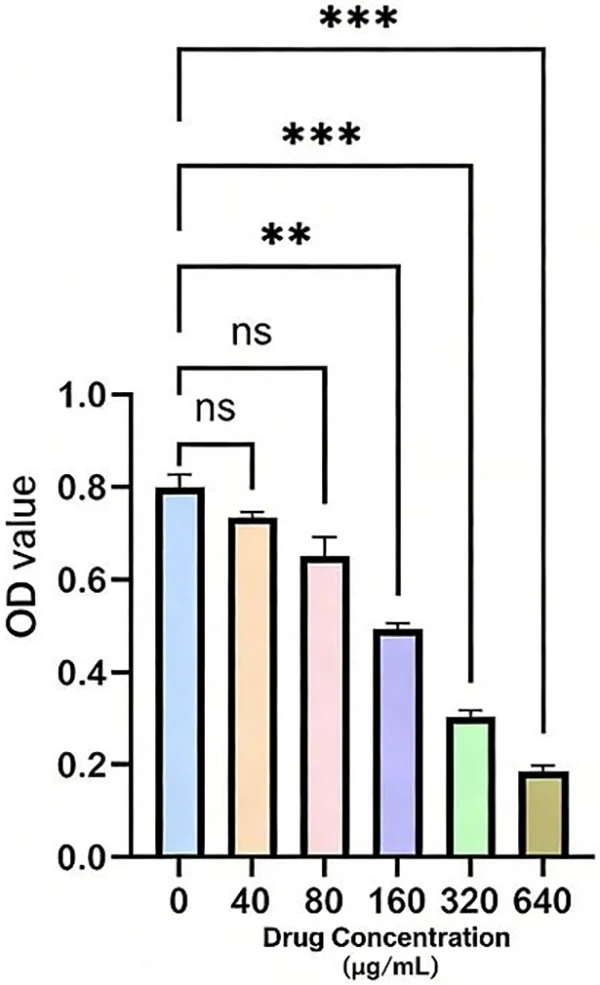
Effect of different concentrations of DMY on mature *P. gingivalis* biofilms. The eradication effect of DMY at 640, 320, 160, 80, and 40 µg/mL on mature *P. gingivalis* biofilms was assessed using crystal violet staining combined with optical density measurement. Compared with the growth control group, the OD values of all treatment groups were decreased, indicating a reduction in *P. gingivalis* biofilm mass, and the extent of reduction was positively correlated with DMY concentration. Compared with the concentration of 0, **P < 0.01, ***P < 0.001.

After treating mature *P. gingivalis* biofilms with different concentrations of DMY for 48 h, the crystal violet staining method was used to evaluate the effect of DMY on mature *P. gingivalis* biofilms. The results showed in Table 6 that compared with the growth control group, the OD values of each experimental group were decreased, i.e., the amount of *P. gingivalis* biofilms was reduced, and the reduction degree was positively correlated with the DMY concentration. Statistical analysis showed that there was no significant difference between the 40 µg/mL, 80 µg/mL experimental groups and the growth control group (*P* > 0.05); while there were statistically significant differences between the 160 µg/mL–640 µg/mL experimental groups and the growth control group (*P* < 0.05) (Figure 6), indicating that 160 µg/mL–640 µg/mL DMY had an eradication effect on mature *P. gingivalis* biofilms. The eradication rate of each experimental group on mature *P. gingivalis* biofilms was calculated, and it was found that with the increase of DMY concentration, the eradication rate on mature biofilms also showed an upward trend. When the DMY concentrations were 40 µg/mL, 80 µg/mL, 160 µg/mL, 320 µg/mL, and 640 µg/mL, the eradication rates were 8.88%, 20.55%, 42.36%, 68.88%, and 85.27%, respectively. Therefore, the MBEC_50_ of DMY against *P. gingivalis* was considered to be 320 µg/mL.

**Table 6:** Effect of different concentrations of DMY on mature *P. gingivalis* biofilms.

Drug concentration (µg/mL)	Mean	Percentile		
		P_25_	P_50_ (median)	P_75_
640	0.186	0.176	0.185	0.196
320	0.304	0.289	0.298	0.318
160	0.495	0.486	0.490	0.505
80	0.652	0.614	0.656	0.689
40	0.736	0.726	0.735	0.744
0	0.800	0.783	0.798	0.828

### 3.3 Effect of DMY on *P. gingivalis* virulence factors

#### 3.3.1 Effect of DMY on the expression of *P. gingivalis* fimbrial genes (*fimA*, *fimR*, *fimS*) and capsular polysaccharide gene *PGN_1525*

**Figure 7:**
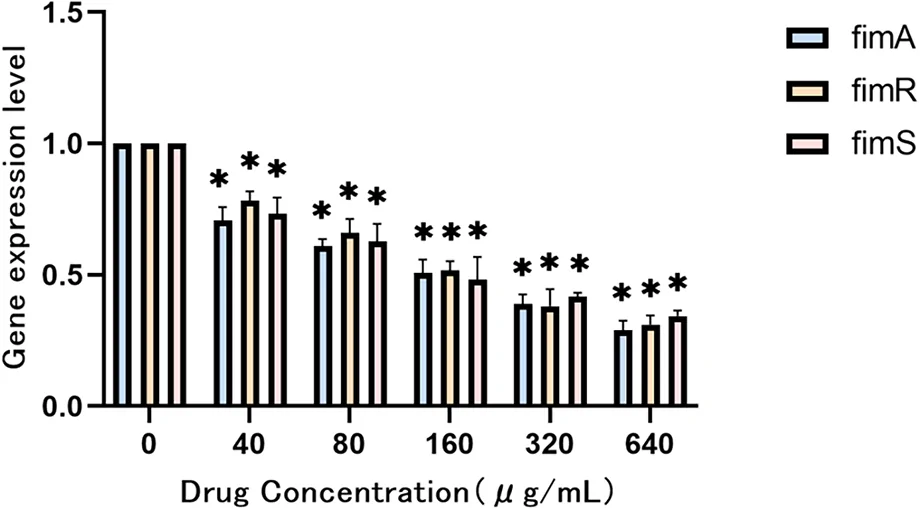
Effect of different concentrations of DMY on the expression of *fimA*, *fimR*, *fimS* genes. The effect of DMY at 640, 320, 160, 80, and 40 µg/mL on the expression of *fimA*, *fimR*, and *fimS* fimbrial genes in *P. gingivalis* was evaluated by qRT-PCR. Compared with the control group, the expression levels of the fimbrial genes *fimA*, *fimR*, and *fimS* in *P. gingivalis* were downregulated to varying degrees in all treatment groups in a concentration-dependent manner. Specifically, the downregulation of gene expression became more pronounced with increasing DMY concentrations. Compared with the concentration of 0, *P < 0.05.

**Figure 8:**
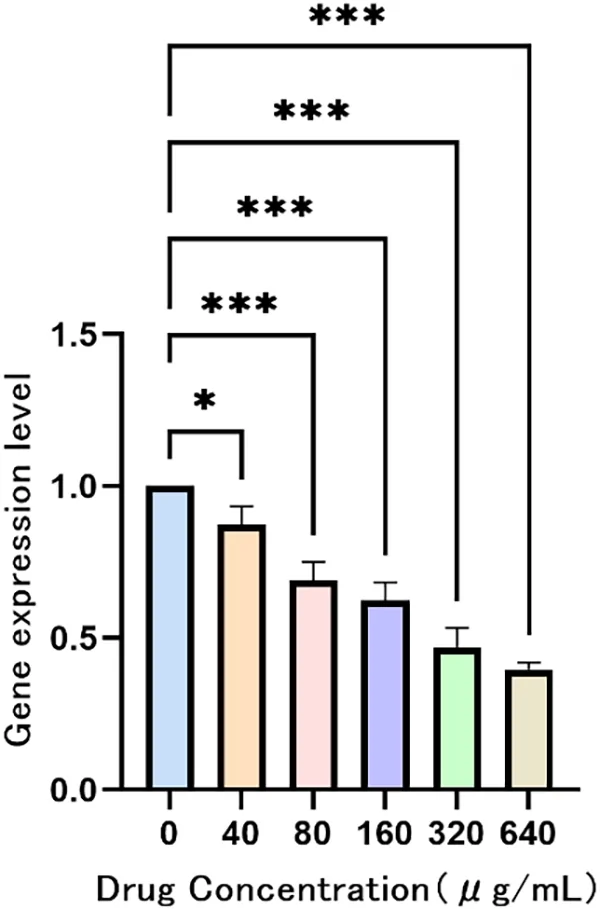
Effect of different concentrations of DMY on the expression of *P. gingivalis* capsular polysaccharide gene *PGN_1525*. The effect of DMY at 640, 320, 160, 80, and 40 µg/mL on the expression of the capsular polysaccharide gene *PGN_1525* in *P. gingivalis* was evaluated by qRT-PCR. Compared with the control group, the gene expression level of capsular polysaccharide *PGN_1525* gradually decreased with increasing DMY concentrations in the treatment groups. Compared with the concentration of 0, *P < 0.05, ***P < 0.001.

The qRT-PCR results showed that under the action of different concentrations of DMY, the gene expression levels of *P. gingivalis fimA*, *fimR*, *fimS*, and capsular polysaccharide *PGN_1525* decreased to varying degrees in a concentration-dependent manner, i.e., the higher the DMY concentration, the more significant the decrease in gene expression levels, with statistically significant differences (*P* < 0.05) (Figures 7, 8). Low DMY concentrations (40 and 80 µg/mL) only induced mild gene downregulation without reaching the 0.5-fold expression reduction threshold; medium and high concentrations (160–640 µg/mL) reduced relative gene expression by over 50% (≤0.5-fold), which meets the biologically meaningful inhibition standard for virulence genes.

#### 3.3.2 Effect of DMY on the proteolytic activity of *P. gingivalis*

**Figure 9:**
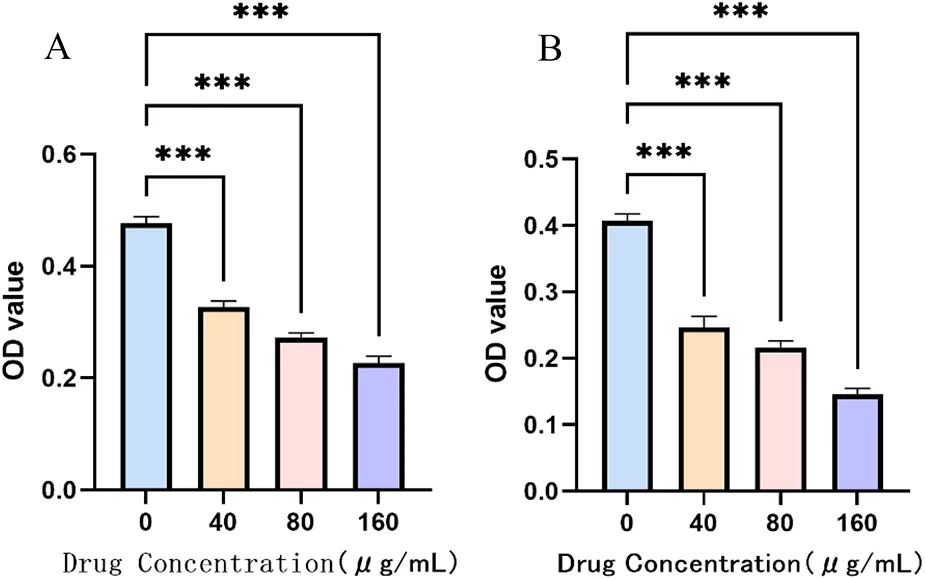
Effect of different concentrations of DMY on Rgp and Kgp. **(A)** The effects of DMY at 40, 80, and 160 μg/mL on the proteolytic activity of Rgp in *P. gingivalis* were determined using a specific protein substrate assay; **(B)** The effects of DMY at 40, 80, and 160 μg/mL on the proteolytic activity of Rgp in *P. gingivalis* were determined using a specific protein substrate assay. The results demonstrated that the proteolytic activities of both Rgp and Kgp in all treatment groups were lower than those in the control group, in a concentration-dependent manner. Compared with the concentration of 0, ***P < 0.001.

The proteolytic activities of Rgp and Kgp in each experimental group (40 µg/mL, 80 µg/mL, 160 µg/mL DMY) were lower than those in the control group, showing a concentration-dependent manner, with statistically significant differences (*P* < 0.05) (Figure 9).

## 4 Discussion

*P. gingivalis* includes various strains, such as ATCC 33277, W83, HG66, W50, etc. (26), which exhibit different virulence and pathogenicity. Among them, ATCC 33277 is collected and preserved by the American Type Culture Collection (ATCC), and its genome has been fully sequenced. It is often used in microbiological experiments, providing abundant data support and references for genomics and proteomics research. ATCC 33277 is the “gold standard” strain of *P. gingivalis*, widely used in scientific research and clinical studies, which can better ensure the reliability and comparability of experimental results. Therefore, this study selected the ATCC 33277 strain for subsequent research.

DMSO is a polar organic solvent, known as the “universal solvent”, which can dissolve a variety of organic and inorganic compounds (27) and has good chemical stability. It is currently widely used in drug screening, cell culture, and microbiological experiments (28). However, studies have shown that DMSO has a certain inhibitory effect on a variety of microorganisms (29, 30), such as *Staphylococcus aureus*, *Streptococcus*, *Escherichia coli*, *Shigella dysenteriae*, etc. It may inhibit bacterial growth by damaging the cell membrane, changing intracellular permeability or cell structure, interfering with cell metabolism, or altering cell hydration and nutrient transport. Therefore, in *in vitro* drug sensitivity tests, the potential impact of DMSO itself on the strain must be considered. The results of this experiment showed that 1% DMSO had no significant effect on the growth of *P. gingivalis*, which was different from the results reported in the literature (29) that DMSO could be safely used as an auxiliary solvent for short-term bacterial growth inhibition tests at concentrations of 3.3%–10%. This may be related to the sensitivity of DMSO to different bacterial species and strains and the different action times of DMSO. The results of this experiment highlight the necessity of fully considering the potential impact of the solvent itself on bacteria before *in vitro* drug sensitivity tests. Although lower concentrations of DMSO may have less impact on the growth of *P. gingivalis*, the lower the concentration of DMSO, the lower the solubility of the drug, which is not conducive to the conduct of subsequent experiments. Therefore, the concentration of DMSO should strike a balance between avoiding impact on bacterial growth and appropriate drug solubility. In summary, 1% DMSO was selected as the drug solvent in subsequent experiments of this study. It is critical to point out that the 1% DMSO solvent selected in this work is only intended for *in vitro* bacterial incubation and cannot be directly adopted for oral mammalian cell experiments.

DMY is a natural flavonoid compound that not only has various biological activities such as antioxidant, anti-inflammatory, and antibacterial effects but also exhibits broad-spectrum antibacterial activity, with clear inhibitory effects on a variety of Gram-positive and Gram-negative pathogenic bacteria (18–22). As the main pathogenic bacterium of chronic periodontitis, *P. gingivalis* exists in the oral cavity mainly in two forms: planktonic cells and biofilms. Planktonic cells refer to *P. gingivalis* that is not attached to teeth or periodontal tissues and is in a free state, usually present in periodontal pockets and saliva, and is the main form of bacterial transmission and diffusion (31). Planktonic cells have strong transmission ability and can spread in the oral cavity through saliva, food residues, or chewing movements, and form new infection foci in periodontal pockets or other parts (32). The results of this study showed that DMY could inhibit the growth of planktonic *P. gingivalis*, with an MIC of 160 μg/mL and an MBC of 320 μg/mL, indicating that DMY could effectively inhibit the growth of *P. gingivalis* at a relatively low concentration and achieve a bactericidal effect at a slightly higher concentration. Compared with the MIC and MBC values of Zanthoxylum bungeanum essential oil against *P. gingivalis* reported by Yi Jinzhi (24) (1 mg/mL and 4 mg/mL, respectively) and the MIC and MBC values of punicalagin against *P. gingivalis* reported by Subinur Arken (2 g/L and 4 g/L, respectively), the flavonoid compound DMY may have more advantages in inhibiting the growth of *P. gingivalis*. Of course, this may be related to the research strains and the concentrations of active ingredients of these natural compounds.

Biofilm is a structure formed by *P. gingivalis* attached to the tooth surface or periodontal pocket wall, composed of bacteria, extracellular polymers, and other biological macromolecules (33). Biofilm destroys periodontal tissues by secreting various enzymes and toxins (such as collagenase, protease, etc.), leading to persistent chronic inflammatory responses, which in turn cause pathological changes such as gingival swelling and bleeding, periodontal pocket formation, and alveolar bone resorption (34). Compared with planktonic cells, bacteria in biofilms have enhanced ability to escape the host immune system by binding to the extracellular matrix. At the same time, due to the physical barrier effect of biofilms, bacteria can better resist the effects of antibiotics and antibacterial therapy, making treatment more difficult (35, 36). Therefore, inhibiting biofilm formation and eradicating mature biofilms can comprehensively reflect the effect of drugs on biofilms. This study found that DMY could inhibit the formation of *P. gingivalis* biofilms and eradicate mature *P. gingivalis* biofilms, with an MBIC_50_ of 160 µg/mL and an MBEC_50_ of 320 µg/mL for *P. gingivalis* biofilms. This result indicates that DMY has a significant effect on inhibiting the formation of *P. gingivalis* biofilms and eradicating mature biofilms, and the MBEC_50_ value is twice the MBIC_50_ value, suggesting that mature biofilms have stronger resistance to DMY. Compared with studies on the effect of other natural plant extracts on *P. gingivalis* biofilms, DMY in this experiment seems to show certain advantages. For example, M. Minami et al. (37) reported that Lonicera caerulea var. emphyllocalyx fruit extract and its component cyanidin-3-O-glucoside had an MBIC_50_ of 178 μg/mL against *P. gingivalis* biofilms, and H. Sulistyani et al. (38) found that roselle calyx extract had an MBIC_50_ of 0.9 mg/mL against *P. gingivalis* biofilms. In contrast, the MBIC_50_ value of DMY is lower. At the same time, studies have shown (25) that epigallocatechin gallate has a significant inhibitory effect on *P. gingivalis* biofilms, with an MBIC_50_ of 7.8 μg/mL, MBIC_90_ of 31.25 μg/mL, and MBEC_50_ of 250 μg/mL, all lower than those of DMY in this experiment, indicating that DMY may be slightly less effective than epigallocatechin gallate in inhibiting biofilm formation and eradicating mature biofilms, but it is still a plant active ingredient with high antibacterial properties.

*P. gingivalis* can secrete a variety of virulence factors, such as fimbriae, gingipains, and capsules, which play a crucial role in the pathogenic process of *P. gingivalis* (39). Fimbriae, as important adhesion factors, mediate bacterial colonization and promote biofilm formation; gingipains promote pathological processes by degrading host tissues and regulating immune responses (40); capsules play an important role in bacterial immune escape and anti-phagocytosis, and also promote bacterial adhesion and biofilm formation (41). Through multiple actions such as adhesion-invasion-immune escape, the three together promote the occurrence and continuous progression of periodontal diseases.

Existing studies have shown that various flavonoid compounds can inhibit the expression and function of important virulence factors such as fimbriae and gingipains of *P. gingivalis*. For example, J Fournier-Larente et al. (11) found that epigallocatechin gallate could downregulate the expression of fimbrial *fimA* and gingipain kgp genes, H Sulistyani et al.'s (38) experiments showed that flavonoids and tannins, the active ingredients of roselle calyx extract, could bind to *P. gingivalis* gingipains and significantly inhibit their activity, and Zhiyan He et al. (42) used real-time fluorescent quantitative PCR technology to prove that quercetin could downregulate the expression of *P. gingivalis* gingipain-related genes rgpA, rgpB, and kgp. In view of this, we speculate that DMY, as a natural flavonoid compound, may also weaken bacterial pathogenicity by inhibiting the fimbriae and gingipains of *P. gingivalis*. Although there are no reports on the inhibitory effect of flavonoid compounds on *P. gingivalis* capsules, our previous experiments have confirmed that DMY can effectively inhibit *P. gingivalis* biofilms, and capsules play an important role in bacterial adhesion, coaggregation, and biofilm formation (41), so we speculate that DMY may also have an impact on capsules.

Fimbriae are curly filamentous protein appendages on the surface of *P. gingivalis* (43), with ultrafine filamentous protrusions extending to the extracellular environment. As key adhesion factors on the bacterial surface, fimbriae endow *P. gingivalis* with strong adhesion ability, enabling it to interact with salivary proteins, extracellular matrix components (such as fibronectin, laminin), eukaryotic cells, and other symbiotic bacteria, helping bacteria achieve initial adhesion to host cells and colonization in periodontal tissues. At the same time, through coaggregation, fimbriae promote the formation and maturation of plaque biofilms. Therefore, the ability of *P. gingivalis* to cause periodontal infections first depends on the expression of fimbriae. The expression and function of *P. gingivalis* fimbriae are regulated by the synergistic action of multiple genes, mainly including structural genes and regulatory genes. *fimA* is the main fimbrial protein gene, responsible for encoding the main structural protein FimA of fimbriae, which is the core subunit of fimbrial assembly and directly affects the morphology and adhesion ability of fimbriae (44). Studies have shown that mutant strains lacking the *fimA* gene have lower ability to invade host cells (45) and impaired biofilm formation (46). *fimR*/*fimS* belongs to the fimbrial regulatory genes of *P. gingivalis*, and their corresponding proteins FimR/FimS are considered to be a two-component regulatory system. Among them, FimS is a transmembrane histidine kinase, which acts as a signal sensor to perceive environmental signals (host microenvironment, temperature, pH value, osmotic pressure, etc.) and transmit the signals to FimR. FimR in the regulatory system is a response regulator protein, which can receive the information transmitted by FimS and further regulate the target gene *fimA*, thereby affecting the synthesis of FimA. Studies have shown that when *fimR*/*fimS* is mutated, the expression level of *fimA* is significantly reduced (47). The results of this study showed that under the action of different concentrations of DMY, the expression levels of *fimA*, *fimR*, and *fimS* of *P. gingivalis* decreased in a concentration-dependent manner, i.e., the higher the DMY concentration, the more significant the decrease in the expression levels of the three fimbrial genes. Therefore, it can be inferred that DMY can downregulate the expression levels of fimbrial genes *fimA*, *fimR*, and *fimS*, thereby possibly reducing the synthesis of *P. gingivalis* fimbriae, weakening its adhesion and coaggregation ability to the host, and ultimately reducing the pathogenicity of *P. gingivalis*.

Gingipains are proteolytic enzymes synthesized in the cytoplasm and secreted extracellularly through transport. They provide approximately 85% of the proteolytic activity of *P. gingivalis* and are considered to be key virulence factors causing periodontal tissue damage (48). Gingipains can be divided into Rgp and Kgp (49), which can specifically recognize and hydrolyze arginine and lysine sites of proteins, respectively. Current studies have shown that *P. gingivalis* can damage host periodontal tissues through multiple pathways relying on the virulence factor gingipains, promoting the pathological process of periodontal inflammation. Firstly, gingipains can directly damage periodontal tissues through their potent proteolytic activity. Literature has confirmed that gingipains can cause the death of different types of host cells (including endothelial cells, human gingival fibroblasts, and osteoblasts, etc.) and lead to the loss of periodontal bone mass (50–53). Experimental results revealed that DMY significantly suppressed the proteolytic activity of gingipain in a concentration-dependent manner. Nevertheless, since DMY was incubated directly with bacterial supernatant, the present assay only verified its direct inhibition against pre-existing extracellular enzyme instead of regulating gingipain synthesis and secretion of *Porphyromonas gingivalis*.

The capsule of *P. gingivalis* is a mucoid polysaccharide structure covering the outer surface of the cell wall, and capsular polysaccharide is its core component. Capsules are highly heterogeneous in antigenicity, and the diversity of their molecular composition and configuration constitutes the basis for serological typing. According to the difference in K antigen, *P. gingivalis* can be divided into six serotypes (K1-K6) (54). As a functional barrier, the capsule plays a very important role between bacteria and the host. Firstly, the capsule can effectively resist the phagocytosis of the host immune system, reduce the recognition and phagocytosis of *P. gingivalis* by phagocytes (such as neutrophils and macrophages), and the more abundant the capsule expression, the stronger its anti-phagocytic ability, thereby enhancing the survival ability of bacteria (55). Secondly, as mentioned earlier, the capsule plays an important role in bacterial adhesion, coaggregation, and biofilm formation. The *PGN-1525* gene, as a key gene encoding capsular polysaccharide, is responsible for synthesizing or modifying specific sugar units in capsular polysaccharide. The expression regulation of this gene is closely related to the growth and pathogenicity of *P. gingivalis* in specific environments (56). This study showed that with the increase of DMY concentration, the expression level of this gene gradually decreased. The inhibition of capsular polysaccharide gene by DMY may lead to structural defects or functional abnormalities of *P. gingivalis* capsule, affecting the adhesion ability of *P. gingivalis*, weakening or losing its immune escape ability, thereby leading to a decrease in its viability and pathogenicity.

The above experimental results indicate that DMY shows clear inhibitory effects on three important virulence factors of *P. gingivalis*—fimbriae, gingipains, and capsules—in terms of related genes or proteolytic activity. This multi-target and multi-dimensional synergistic effect may be one of the important mechanisms by which DMY effectively inhibits planktonic *P. gingivalis* and biofilms.

Of course, this study also has certain limitations. First, this experiment only used the standard strain ATCC 33277 of *P. gingivalis* as the research object, and did not include clinical isolates and drug-resistant mutant strains from different geographical sources and with different genetic backgrounds. Second, this study did not set up clinically commonly used anti-periodontal pathogenic drugs such as chlorhexidine and metronidazole as positive controls, so it was impossible to clarify the advantages and disadvantages of the antibacterial effect of DMY through comparison, nor was it easy to objectively evaluate its potential value in clinical application. Third, this study only initially detected the changes of some virulence factors at the gene expression level or protein activity level, and did not carry out systematic mechanism exploration. It was impossible to clarify the specific molecular pathway by which DMY kills *P. gingivalis* cells, and it was difficult to fully reveal the complex mechanism of its antibacterial effect, which requires a lot of continuous exploration in the future. Fourth, crystal violet staining was used to quantify total biofilm biomass in this study. This assay stains all biofilm components including viable bacteria, dead cell debris and extracellular matrix, and cannot specifically quantify viable bacterial counts within biofilms. Therefore, our results only reflect the inhibitory effect of the tested compound on overall biofilm biomass rather than the exact changes in viable bacterial abundance inside biofilms.Fifth, this study did not carry out relevant safety tests of DMY on host oral-related cells (such as gingival fibroblasts, periodontal ligament cells, oral epithelial cells, etc.); at the same time, the *in vitro* cell model used in this study was different from the real *in vivo* physiological and pathological environment, and the pharmacokinetic characteristics, effective dose and safety of DMY *in vivo* remain to be further explored. In view of the above limitations, future research will focus on the following aspects: first, expand the scope of research strains, include *P. gingivalis* clinical isolates and drug-resistant mutant strains from different geographical sources and with different genetic backgrounds, to verify the universality of the antibacterial effect of DMY; second, add positive controls such as chlorhexidine and metronidazole to clarify the difference in antibacterial effect between DMY and clinically commonly used drugs; third, further explore the molecular mechanism of DMY's antibacterial effect, and clarify the specific pathway of its inhibition on *P. gingivalis* growth and biofilm formation through relevant experiments such as membrane permeability, ROS production, DNA integrity and protein denaturation; fourth, improve the biofilm characterization methods, and comprehensively evaluate the inhibitory effect of DMY on biofilms by combining CLSM, SEM and matrix component detection technologies; fifth, carry out host cell safety tests to clarify the toxic effect of DMY on normal oral cells, and at the same time construct a chronic periodontitis animal model to carry out *in vivo* experiments, systematically evaluate the *in vivo* therapeutic effect, pharmacokinetic characteristics and long-term use safety of DMY, so as to provide more sufficient and powerful experimental evidence for the clinical application of DMY in the treatment of chronic periodontitis.

Systemic oral or intravenous DMY delivery requires excessively high systemic doses to reach therapeutically effective subgingival concentrations, accompanied by high risks of systemic cytotoxicity and low target tissue bioavailability. In contrast, local subgingival administration (including sustained-release periodontal gel, subgingival dressing and short-term therapeutic mouth rinse) is the optimal clinical strategy. Local application concentrates DMY within isolated periodontal anaerobic pockets at 160–640 µg/mL, with minimal systemic absorption. Short-term mucosal contact (≤48 h) at these concentrations avoids irreversible epithelial cell damage, and sustained-release formulations can further lower peak DMY exposure to balance efficacy and biosafety.

Combining all *in vitro* phenotypic data, DMY exerts synergistic anti-*P. gingivalis* effects via multiple pathways: (1) direct bacteriostatic and bactericidal activity against planktonic bacteria; (2) dual functions of inhibiting *de novo* biofilm formation and eradicating mature biofilm biomass; (3) transcriptional suppression of adhesion-related virulence genes (*fimA*, *fimR*, *fimS*, *PGN_1525*) to block fimbria and capsule synthesis; (4) direct extracellular inhibition of Rgp and Kgp gingipain proteolytic activity to reduce host tissue degradation. Consistent with published flavonoid antibacterial mechanisms, DMY may also interfere with bacterial membrane integrity and intracellular metabolism to amplify its antimicrobial potency.

Published MIC data of DMY against common pathogens provide a reference to evaluate the intrinsic susceptibility of *P. gingivalis*. The reported MIC range of DMY is 32–128 µg/mL for *S. aureus*, 128–256 µg/mL for *E. coli*, and 64–256 µg/mL for *C. albicans*. The MIC of DMY against *P. gingivalis* (160 µg/mL) falls within the medium susceptibility range of DMY-targeted pathogens, demonstrating that *P. gingivalis* does not possess stronger intrinsic resistance than typical oral and cutaneous microorganisms.

Existing *in vitro* cytocompatibility studies confirm that DMY concentrations ≤80 µg/mL induce no detectable cytotoxicity in human gingival fibroblasts and oral epithelial cells under long-term incubation. Concentrations of 160–320 µg/mL DMY only cause mild, reversible cellular damage under continuous prolonged culture. The MIC (160 µg/mL) and MBC (320 µg/mL) measured in this study fit the safe short-term exposure window for local periodontal treatment. To eliminate cytotoxic risks for long-term clinical use, sustained-release local delivery formulations should be developed to reduce peak DMY concentration and shorten epithelial contact time.

## 5 Conclusion

The results of this study show that DMY exhibits significant antibacterial activity against both planktonic and biofilm forms of *P. gingivalis*. It can not only inhibit the proliferation of planktonic *P. gingivalis* but also effectively inhibit the formation of its biofilms and eradicate mature biofilms. In addition, DMY can significantly downregulate the expression levels of *P. gingivalis* fimbriae-related genes (*fimA*, *fimR*, *fimS*) and capsular polysaccharide gene *PGN-1525*, and inhibit the proteolytic activity of gingipains Rgp and Kgp. DMY is expected to be used as a new plant-derived antibacterial agent or antibacterial active ingredient in the field of *P. gingivalis* infection control and periodontal disease prevention and treatment. Further in-depth mechanism research and *in vitro* and *in vivo* verification are still needed in the future to further improve this conclusion and provide a basis for clinical application.

## Data Availability

The datasets presented in this study can be found in online repositories. The names of the repository/repositories and accession number(s) can be found in the article/Supplementary Material.
